# *Toxoplasma gondii* in Australian macropods (*Macropodidae*) and its implication to meat consumption

**DOI:** 10.1016/j.ijppaw.2021.09.004

**Published:** 2021-09-14

**Authors:** Yannick Borkens

**Affiliations:** College of Public Health, Medical and Veterinary Science, James Cook University, Townsville, Queensland, 4811, Australia

**Keywords:** Australian macropods, Food-borne pathogens, Hunting, Meat inspection, One health, *Toxoplasma gondii*

## Abstract

*Toxoplasma gondii* is a worldwide occurring apicomplexan parasite. Due to its high seroprevalence in livestock as well as in game animals, *T*. *gondii* is an important food-borne pathogen and can have significant health implications for humans as well as for pets. This article describes the prevalence of *T*. *gondii* in free-ranging macropods hunted for consumption. All hunted macropod species (commercial as well as non-commercial hunt) show a positive seroprevalence for *T*. *gondii*. This seroprevalence is influenced by various factors, such as sex or habitat. Furthermore, the parasite shows a high level of genetic variability in macropods. Genetically variable strains have already caused outbreaks of toxoplasmosis in the past (Canada and the US). These were attributed to undercooked game meat like venison. Despite this risk, neither Australia nor New Zealand currently have food safety checks against foodborne pathogens. These conditions scan pose a significant health risk to the population. Especially, since cases of toxoplasmosis have already been successfully traced back to insufficiently cooked kangaroo meat in the past.

## Highlights

• The kangaroo hunt is an important industry in Australia.
• 7 species of kangaroos and wallabies are hunted for commercial purpose (for human and pet consumption).
• Food security checks against foodborne pathogens (including *T*. *gondii*) are not a requirement of the Australia New Zealand Food Standard Code.
• The databases Medline, Web of Science, SCOPUS and Informit were used.
• 6 scientific publications were reviewed in this publication.

## Graphical abstract

**Figure:**
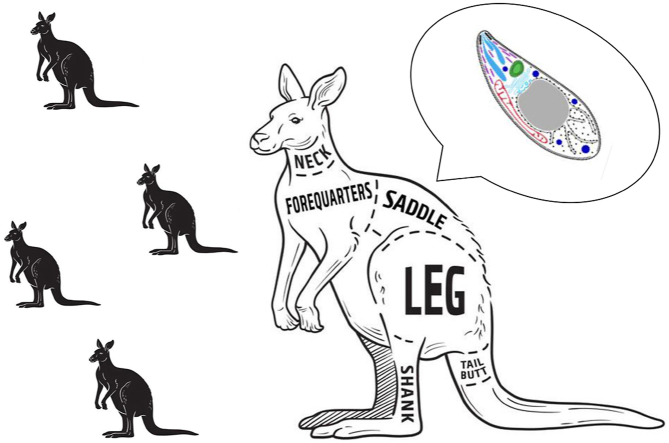

## 1 Introduction

*Toxoplasma gondii* is a protozoan parasite found across the world (Storch and Welsch, 2014; Kochanowsky and Koshy, 2018; Stock, 2020). While the definitive hosts of *T*. *gondii* are members of the feline family, all warm-blooded animals (birds and mammals, including humans) function as intermediate hosts (Kochanowsky and Koshy, 2018). *T*. *gondii* causes the disease toxoplasmosis, which is often associated with flu-like symptoms including swollen lymph nodes, muscle aches and pain (Kochanowsky and Koshy, 2018). While most infected people are asymptomatic, those who are pregnant or have a compromised immune system can develop significant health issues.

### 1.1 Acute and congenital toxoplasmosis

The acutely acquired form of toxoplasmosis is called acute or primary toxoplasmosis. Due to its flu-like manifestation, the correct diagnosis of acute toxoplasmosis can be difficult (Dubey, 2010b; Kochanowsky and Koshy, 2018). A more severe form of the disease is secondary or congenital toxoplasmosis. If women become infected during pregnancy, *T*. *gondii* can be passed transplacentally to their foetuses resulting in congenital toxoplasmosis (Dubey, 2010c). Congenital toxoplasmosis can cause miscarriage of the foetus, still birth- or serious health consequences in the new born baby (McAuley, 2014; Peyron et al., 2017). However, vertically transmission without symptomatic presentation is also possible (Chaudry et al., 2014). Especially in developing countries, congenital toxoplasmosis is a serious threat to the unborn foetus (Dubey, 2010c). Acute toxoplasmosis can develop into a chronic disease. This form is described as latent toxoplasmosis. The majority of infections are asymptomatic (Dubey, 2010b).

### 1.2 Epidemiology

As one of the most common parasitic infections in humans worldwide, *T. gondii* can be found in low- and middle-income countries as well as in high-income countries. In Australia, the prevalence of latent toxoplasmosis in women of childbearing age is 23% (n = 308) (Flegr et al., 2014). This prevalence is lower than the prevalence in other high-income countries like France (54%; n = 13459), Germany (63%; n = 4854) or New Zealand (35%; n = 500) (Flegr et al., 2014). In the United States, the prevalence in women of childbearing age is lower at only 9% (n = NA) (Flegr et al., 2014). In total, 14% of people aged 12 to 49 show a positive seroprevalence in the US (Robert-Gangneux and Dardé, 2012). Examples for developing countries with a very high seroprevalence in women are Madagascar (84%; n = 599), Nigeria (78%; n = 352) and Cameroon (77%; n = 1014) (Flegr et al., 2014). Although it appears that the prevalence of *T*. *gondii* is not influenced by the climate (Jensen et al., 2010; Simon et al., 2013), warm climates and low-lying areas seems to favour the infection (Dubey, 2010a).

### 1.3 Route of infection

There are several ways that humans can become infected with *T*. *gondii*. Infection via the faecal-oral route by ingestion of infective oocysts with contaminated water or undercooked vegetables is common (Flegr et al., 2014). The meat of infected animals contains cysts with bradyzoites. Humans can become infected after consumption of undercooked meat (especially pork, lamb and venison; transmission through beef is very rare) (Dubey, 1998; Flegr et al., 2014; Blaga et al., 2019). Infection can also occur after the consumption of animal milk containing tachyzoites of *T*. *gondii* (Flegr et al., 2014; Mofokeng et al., 2020). Infections via blood donations or organ transplants are also possible (Flegr et al., 2014; Khurana and Batra, 2016). Furthermore, vertical transmission is also possible. This happens when the mother becomes infected during pregnancy. Vertical transmission can also occur if the toxoplasmosis of a chronically infected woman is reactivated in the course of an immunosuppressive disease, or if seroconversion occurs in an immunocompetent woman only a few months before conception (Kodjikian et al., 2004). Vertical transmission can be asymptomatic (Chaudry et al., 2014).

As the definitive host of *T*. *gondii*, cats play an important role in the lifecycle of *T*. *gondii* as well as its maintenance in nature (Dabritz and Conrad, 2010). Infections in cats occur horizontally by ingestion of tissue of intermediate hosts as well as vertically (Atmaca et al., 2013; Calero-Bernal and Gennari, 2019). Inside the cat intestine, *T*. *gondii* undergoes sexual reproduction that results in the production of oocysts. The quality of oocyst produced varies between 3 and 810 million per cat infection (Dabritz and Conrad, 2010). The oocysts will be shed with faeces in the environment; there they can survive from several months up to one year (Dabritz and Conrad, 2010). Thus, cats are able to spread many oocysts widely, which in turn increases the likelihood of infecting intermediate hosts. Chronically infected cats, however, show a certain resistance due to T. gondii antibodies and shed oocysts more seldomly (Dubey, 1976). There are several risk factors for domestic cats to contract *T*. *gondii*. Cats fed with fresh or wet food show a higher seroprevalence than cats fed with dry food (Yücesan et al., 2019). For this reason, infection can be prevented by feeding cats with dry, canned or boiled food (Frenkel and Dubey, 1972). A further risk factor are cats, both domestic or feral, who roam in the environment. These cats are more likely to become infected with *T*. *gondii* as a result of praying on small mammals, scavenging on carcasses and ingestion of oocysts (Gerhold and Jessup, 2013; Shapiro et al., 2019; Brennan et al., 2020). To reduce that risk, pet cats should be deterred from hunting (Frenkel and Dubey, 1972). Cat owners are at risk from close contact with cats and their faecal material (Flegr et al., 2014; Pleyer et al., 2019; Yücesan et al., 2019).

### 1.4 T. gondii as a food-borne pathogen

All warm-blooded animals can function as an intermediate hosts for *T*. *gondii*, including wild and domestic birds, smaller animals such as rodents as well as Australian monotremes and marsupials, livestock species, larger predators such as dogs and bears and marine mammals such as dolphins and whales (Gamble, 1997; Munday et al., 1998; Jensen et al., 2010; Tryland et al., 2013; Bezerra et al., 2015; Cano-Terriza et al., 2016; Dubey et al., 2016; Hillman et al., 2016; Reiterová et al., 2016; San Miguel et al., 2016; Verma et al., 2016; Shokri et al., 2017; Iqbal et al., 2018; Rasambainarivo et al., 2018; Cerqueira-Cézar et al., 2019). For this reason, *T*. *gondii* is one of the most relevant food-borne pathogens. High prevalence of infection with *T*. *gondii* has been found in poultry, pigs, sheep and goats, among others (Gamble, 1997; Shokri et al., 2017; Ducournau et al., 2020; Mofokeng et al., 2020). Cattle, on the other hand, show a certain resistance (Blaga et al., 2019; Mofokeng et al., 2020). However, the seroprevalence depends heavily on different husbandry conditions. These factors include indoor or outdoor rearing, herd size, farming with different species, feeding with seasonal foods without chemical processing, and whether cats are present on farms (Bawm et al., 2016; Djokic et al., 2016).

In addition to livestock animals, high prevalence were also found in game species. These include wild boars (*Sus scrofa*) (Matsumoto et al., 2011; Jeong et al., 2014; Jokelainen et al., 2015; Cleveland et al., 2017; Roqueplo et al., 2017; Laforet et al., 2019; Machado et al., 2019; Bier et al., 2020; Kornacka et al., 2020; Sgroi et al., 2020) and various deer species (*Cervus* spp., *Odocoileus* spp., *Alces alces*) (Matsumoto et al., 2011; Olamendi-Portugal et al., 2012; Dubey et al., 2014; Tavernier et al., 2015; Rocchigiani et al., 2016; Remes et al., 2018; Kolören et al., 2019; Bier et al., 2020). *T*. *gondii* has been found in these species on all continents. Outbreaks of toxoplasmosis have been associated with the consumption of undercooked game meat in Australia (Kangaroo) as well as in the United States and in Canada (Deer) (Robson et al., 1995; Gaulin et al., 2020; Schumacher et al., 2020; Westling, 2020). For this reason, hunters, people, who engage in hunting trips as well as livestock and farm workers, are people at risk (Muñoz-Zanzi et al., 2013; Machado et al., 2019; Stelzer et al., 2019; Westling, 2020). Furthermore, *T*. *gondii* can also cause economic damage. For example, due to the reduced health of farm animals or the lack of workers who stay away from work because of toxoplasmosis (Stelzer et al., 2019).

### 1.5 Kangaroo hunt in Australia

In Australia, macropods (kangaroos and wallabies) are a major burden for Australia's ecology and livestock industry due to their high number and their impact on grazing land (Wilson and Edwards, 2019). The increase in certain populations is due to an oversupply of food sources, provided by agriculture. As a result, the populations become an ecological burden (Kenyon, 2019; Wilson and Edwards, 2019). To reduce that burden, Australia allows certain kangaroos species to be hunted and the meat used for human and pet consumption. These species are specified in the official hunting code. Due to its nutritional value as well as the environmental benefits of reducing population numbers, the human and animal consumption of kangaroo meat is gaining popularity (Sinclair et al., 1987; Bhattacharya et al., 2006; Wilson and Edwards, 2019; Classen et al., 2020). This leads to concerns about how rigorously kangaroo meat is checked given the possibility it can be a source of infection with *T*. *gondii* for humans and pet animals. As grazing herbivores, kangaroos can easily ingest oocytes shed by cats and become infected. Because the meat can be easily purchased from many suppliers and because cats love the meat, unchecked kangaroo meat can be an important source of infection for cats in Australia. Thus, infected cats, through shedding of millions of oocysts in the environment, can be a source of infection for humans and other species. A more comprehensive understanding of the prevalence of *T*. *gondii* in Australian macropods and the way their meat is checked for this parasite could provide important information related to risks posed for human and pet health.

## 2 Methodology

Four scientific databases were searched systematically for literature. The search followed the five-step approach recommended by Arksey and O'Malley (2005). The used search terms were defined by using the Medical Subject Headings (MeSH) provided by Medline (Baumann, 2016).

### 2.1 Search strategy

A comprehensive search of peer-reviewed published literature was conducted on September 1st, 2020. A combination of medical search terms, separated in three groups, were used to search the databases Medline, Web of Science, SCOPUS and Informit. The used search terms are listed in section 2.2 Search Terms.

A search of grey literature was used to gain additional information not covered by the scientific literature and to fill knowledge gaps. This includes official government publications such as official codes or laws, but also broadcast and news publications. The search engine Google was used to search specifically for grey literature.

### 2.2 Search terms

The search terms used in this review are divided into three search groups: *Microbe*, *Host* and *Food Safety and Food Security*. The first search group unites all terms about the microbe as well as the disease. The second search group unites all terms about the host. This group includes the specific search terms *macropods* and *marsupials*. The search term *Australia* specifies the geographic area and ensures that studies without a focus on Australia are excluded. Last but not least, the third search group unites all terms about food handling and food security. A detailed list of all search terms used is given in Table 1.

**Table 1:** The following search terms were used to search for literature. The terms were identified using the Medical Subject Headings (MeSH) thesaurus, produced by the National Library of Medicine. The terms have been organised into three groups to make understanding straightforward.

Group one: Microbe	
*Toxoplasma gondii*	toxoplasma × OR “toxoplasma gondii” OR “toxoplasma gondius” OR “toxoplasma hominis”
Toxoplasmosis	toxoplasmosis OR “infection, toxoplasma gondii” OR “toxoplasma gondii infection” OR “animal toxoplasmosis” OR “animal toxoplasmoses” OR congenital toxoplasma infection × OR congenital toxoplasma gondii infection × OR congenital toxoplasmosis OR congenital toxoplasmoses OR fetal toxoplasmosis OR fetal toxoplasmoses
**Group two: Host**	
Kangaroos	kangaroo × OR Macropodidae OR Macropod × Or petrogale × OR quokka × OR wallaby × OR wallaroo*
Marsupials	marsupial × OR “methateria”
Wild Animals	feral animal × OR nondomestic animal × OR nondomesticated animal × OR stray animal × OR wild animal*
Australia	australia/OR “australian” OR australian capital territory/or new south wales/OR northern territory/OR queensland/OR south australia/OR tasmania/OR victoria/OR western Australia/
**Group three: Food Safety and Food Inspection**	
Food Safety	“food safety” OR “safety, food” OR food adulteration × OR food contamination × OR food parasitology/
Food Handling	“food handling” OR “food processing” OR “meat packing industry” OR “meat-packing industry” OR pasteurization × OR ultrapasteurization*
Meat Market	“meat trade” OR meat/OR red meat/
Hunting	“hunting” OR “hunt” OR “animal hunt”

### 2.3 Study criteria and selection

**Fig. 1:**
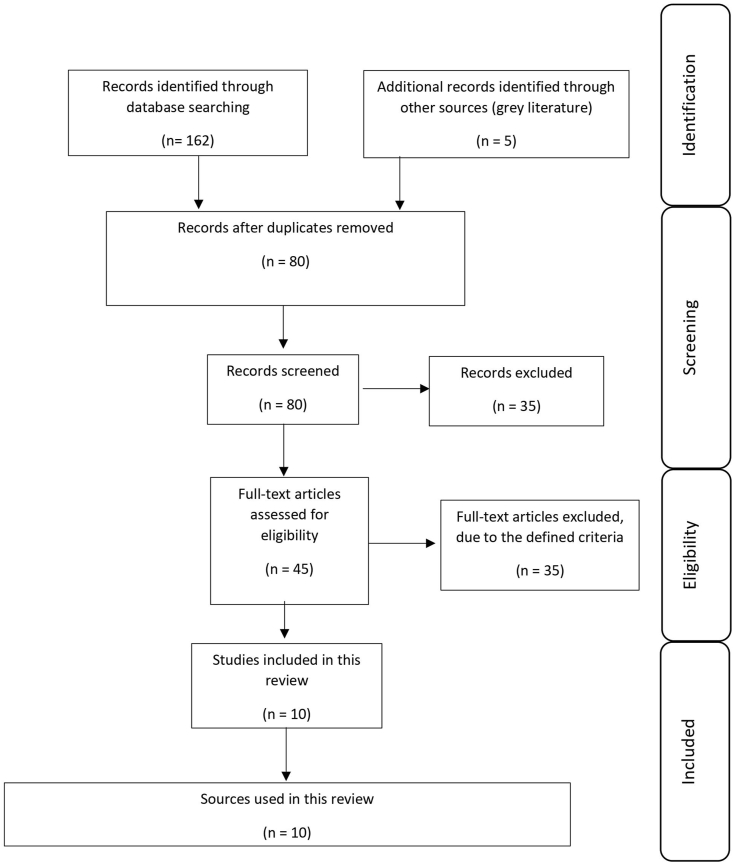
Flowchart of article selection.

To identify eligible publications, inclusion and exclusion criteria were defined. The criteria are displayed in Table 2. After the search was conducted, duplicated publications listed in the databases were removed. This was followed by the screening of the titles and abstracts, further removing ineligible publications. After ineligible publications were excluded, the remaining publications were subjected to a full-text review. Full-text articles that did not meet the defined criteria were finally excluded. Fig. 1 shows the flow diagram for the selection of publications. This flow diagram was created following the Preferred Reporting Items for Systematic reviews and Meta-Analyses (PRISMA) recommendations (Tricco et al., 2018) and includes the inclusion of six peer reviewed papers and four grey literature sources.

**Table 2:** Inclusion and exclusion criteria defined to identify publications that are eligible for review.

Inclusion Criteria	Exclusion Criteria
Articles, which are relevant for this literature review should be/have …	Articles, which are relevant for this literature review should not be/have …
➢ Published in peer-reviewed journal ➢ Original research paper (or) ➢ Review paper ➢ Full-text available; complete downloadable (open access or access via university/library network) ➢ A study focus specifically related to kangaroos ➢ A study focus specifically related to the trade of kangaroo meat (pet and human market) ➢ A study focus specifically related to Australia ➢ Published in English	➢ A limited access; full-text is not available and/or downloadable ➢ A study focus which is not specifically related to kangaroos or other marsupials ➢ A study focus which is not specifically related to the meat trade or living animals ➢ Published in a language other than English

## 3 Results

A total of 162 peer-reviewed publications and 5 additional records met the eligibility criteria (Fig. 1). After duplicated studies were removed, the number was reduced to 80 publications. These 80 studies were screened for title, followed by a review of the abstract. This leads to a reduction to 45 publications. These 45 publications were eligible for the full-text review and were reviewed taking the defined inclusion and exclusion criteria into account. This full-text review resulted in 6 scientific publications for use in this review and four grey literature documents.

Five of these publications set focus on the seroprevalences of various kangaroo species (Parameswaran et al., 2009a,b; Mayberry et al., 2014; Hillman et al., 2016; Taggart et al., 2019). In addition, two elaborate on the implications of reproduction (Parameswaran et al., 2009b; Mayberry et al., 2014). One publication deals with the genetic diversity of *T*. *gondii* infections in macropods (Pan et al., 2012).

Grey literature, described as *evidence not published in commercial publications* by Paez (2017), were used to extract information and data that could not be found in official publications. In this review, the *National Codes of Practice for the Humane Shooting of Kangaroos and Wallabies* were used to provide more information. Since grey literature is usually not subjected to peer review, the grey publications were not used on an equal footing as identified publications; their role was to fill information gaps.

## 4 Discussion

The high susceptibility to *T*. *gondii* of Australian macropods has often been verified by different studies and case reports (Parameswaran et al., 2009a; Hillman et al., 2016). The majority of these studies describe animals in captivity (Hillman et al., 2016). Studies on macropods infected with *T*. *gondii* in captivity report severe pathological lesions and signs including sudden death (Reddacliff et al., 1993; Moré et al., 2010). However, the literature on *T*. *gondii* in feral macropods is limited. Furthermore, this literature review revealed a lack of scientific literature in regard to risk of consumption of kangaroo meat for human and pet health.

### 4.1 Seroprevalence in Australian macropods

The seroprevalence of *T*. *gondii* in Australian macropods is described in several publications. However, the main objectives as well as the methodology used differ in each study. In general, Australian macropods show a prevalence of *T*. *gondii* that is influenced by several factors (Parameswaran et al., 2009a; Taggart et al., 2019). In addition, the various *T*. *gondii* strains observed in infected macropods show a genetic diversity (Pan et al., 2012). Those genetically diverse strains are able to infect several organs of the same host independently. However, multiple organ infections are also possible (Pan et al., 2012).

Although the seroprevalence of Australian macropods depends on several factors, there is no significant difference in prevalence between the large kangaroos and wallabies (Taggart et al., 2019). In kangaroos, the detected seroprevalence of *T*. *gondii* in male kangaroos are significantly lower than in female kangaroos (Parameswaran et al., 2009a; Taggart et al., 2019). These differences agree with other studies which describe a higher prevalence in female than in male livestock (sheep and goats) and are due to sex specific behaviour and diets (Parameswaran et al., 2009a). However, a sex specific difference in wallabies was not detected (Taggart et al., 2019). A change in the reproductive performance due to a *T*. *gondii* exposure has also not been observed (the species examined in the study was *Macropus fuliginosus*) (Mayberry et al., 2014). Another factor that affects the seroprevalence is the location and whether the host population is located on the mainland or on islands. Taggart et al. described, that the *T*. *gondii* seroprevalence in kangaroos (the species examined in the study was again *M*. *fuliginosus*) from Kangaroo Island (South Australia) is significantly greater than in kangaroos from the direct adjacent mainland. They conclude, that this difference is due to a difference in ecological factors such as cat density, feeding ecology, climate or soil characteristics (Taggart et al., 2019).

Vertical transmission of *T*. *gondii* in macropods was also observed. Parameswaran et al. investigated the vertical transmission of *T*. *gondii* in 2009 and demonstrated that all seropositive dams also had seropositive pouch young. Seronegative dams on the other hand only had seronegative pouch young. Since it was highly unlikely that the pouch young were exposed to *T*. *gondii* oocysts in the environment, Parameswaran et al. concluded that all pouch young were infected with *T*. *gondii* vertically. They used both an Enzyme-linked Immunosorbent Assay (ELISA) as well as a nested Polymerase Chain Reaction (PCR). The PCR confirmed the serological results (Parameswaran et al., 2009b).

While all hunted kangaroo species show a high seroprevalence for *T*. *gondii*, this seroprevalence vary markedly (Hillman et al., 2016). Some publications discuss hypotheses for these variations, such as cat density and environmental factors. However, there are a number of limitations in the studies making any firm conclusion impossible (Taggart et al., 2019). This includes small sample sizes in some studies and issues regarding, selection biases, especially when the examined kangaroos are not collected randomly (Taggart et al., 2019).

### 4.2 Macropods hunted for the human consumption

The consumption of kangaroo meat is becoming increasingly popular due its leanness and healthy fats (Sinclair et al., 1987). Australia benefits from professional kangaroo hunting both economically and ecologically (Wilson and Edwards, 2019). However, there is a potential risk for outbreaks associated with food-borne pathogens, especially because kangaroo meat is often consumed raw (Parameswaran et al., 2009a).

A seropositive prevalence of *T*. *gondii* was observed in the macropod species hunted for the human consumption (Parameswaran et al., 2009a; Pan et al., 2012; Mayberry et al., 2014; Hillman et al., 2016; Taggart et al., 2019). This seroprevalence is associated with various factors. Female kangaroos, for example, show a higher prevalence than males (Parameswaran et al., 2009a; Taggart et al., 2019). This sex difference is not reported in wallabies (Taggart et al., 2019). The reason for this is unclear, but it is likely that this variance is due to sex differences in the eating behaviour (Parameswaran et al., 2009a). Female kangaroos are able to crop shorter grass better than males, which are therefore forced to find other food sources (Newsome, 1980; Parameswaran et al., 2009a). For this reason, females are more likely to be exposed to possible soil infection with *T*. *gondii* than males (Parameswaran et al., 2009a). This difference also increases the risk of vertical transmission from mothers to their young (Parameswaran et al., 2009b). Geographic locations appear to also have an influence on seroprevalence. Macropod populations on islands show a higher prevalence than populations on the mainland. Possible reasons could relate to feeding behaviour or domestic and feral cat density (Taggart et al., 2019). Animals in metropolitan and leisure areas such as golf courses also show a higher seroprevalence than animals in nature reserves (Mayberry et al., 2014). This is important, because the majority of kangaroos are hunted on pastoral grounds and other locations close to human populations and not in remote areas (Wilson and Edwards, 2019).

A search of the grey literature identified the official codes for kangaroo hunting, published and endorsed by the Natural Resource Management Ministerial Council. Two codes exist in Australia: *The National Code of Practice for the Humane Shooting of Kangaroos and Wallabies for Commercial Purpose* and the *National Code of Practice for the Humane Shooting of Kangaroos and Wallabies for Non-Commercial Purpose*. Hunting for the food market is defined in the commercial code, including the species intended for hunting. Five species are earmarked for the commercial hunt: *Macropus giganteus*, *Macropus fuliginosus*, *Macropus rufus*, *Macropus rufogriseus* and *Thylogale billardierii*. However, not all species are hunted in all states or territories. The code defines commercial purposes as “where the kangaroo or wallaby is shot to be used as a product (carcass or skin) to be sold within Australia or overseas”. In addition to these five species, two more species are earmarked for non-commercial hunting: *Macropus agilis* and *Macropus parryi*. Both codes are freely available online (Director of Wildlife Trade Assessments, 2008; AgriFutures Australia, 2020). Table 3 gives an overview of the hunted species and their recorded seroprevalence for *T*. *gondii*.

**Table 3:** Overview of all macropod species (kangaroos as well as wallabies), hunted for commercial and non-commercial purpose as well as their seroprevalence for *T*. *gondii* and locations.

Species	Hunted in State or Territory	Reported seroprevalences	Origin of Sample	Year of Sampling	Detection Method	Reference	Access
*Macropus giganteus* (Eastern Grey Kangaroo)	Hunted in NSW and QLD for commercial purpose	0% (0/4)	Roma, Queensland	No specific time	Mouse bioassay (suspension of host brain; injected SC; mouse brain emulsified and examined for *T*. *gondii* cysts)	Smith and Munday, (1965)	https://doi.org/10.1111/j.1751-0813.1965.tb06562.x
		0% (0/3)	Blackall, Queensland	No specific time	Mouse bioassay	Smith and Munday, (1965)	https://doi.org/10.1111/j.1751-0813.1965.tb06562.x
		0% (0/112)	Roma, Queensland	2004	ELISA	Hillman et al. (2016)	https://doi.org/10.1016/j.ijppaw.2015.12.002
				–			
				2005			
		3.1% (2/65)	Sydney, NSW	2006	ELISA	Hillman et al. (2016)	https://doi.org/10.1016/j.ijppaw.2015.12.002
*Macropus fuliginosus* (Western Grey Kangaroo)	Hunted in NSW, SA and WA for commercial purpose	15.5% (33/219)	Perth, Western Australia	No specified time; 18 month period	ELISA;	Parameswaran et al. (2009a)	https://doi.org/10.1016/j.parint.2009.01.008
					Modified Agglutination Test;		
					Polymerase Chain Reaction		
		100% (5/5)	Menzies, Western Australia	2008	Multi-locus PCR-DNA sequencing (tissue samples)	Pan et al. (2012)	https://doi.org/10.1371/journal.pone.0045147
		15% (7/47)	Thompson Lake Nature Reserve, Perth, Western Australia	05.2006	Indirect fluorescence antibody test (blood samples)	Mayberry et al. (2014)	https://doi.org/10.7589/2013-03-064
				– 10.2008			
		8% (2/24)	Harry Waring Marsupial Reserve, Perth,	05.2006	Indirect fluorescence antibody test (blood samples)	Mayberry et al. (2014)	https://doi.org/10.7589/2013-03-064
				–			
				10.2008			
		13% (2/15)	Melville Glades Golf Club, Perth, Western Australia	05.2006	Indirect fluorescence antibody test (blood samples)	Mayberry et al. (2014)	https://doi.org/10.7589/2013-03-064
				–			
				10.2008			
		56% (9/56)	Marangaroo Golf Course, Perth, Western Australia	05.2006	Indirect fluorescence antibody test (blood samples)	Mayberry et al. (2014)	https://doi.org/10.7589/2013-03-064
				–			
				10.2008			
*Macropus rufus* (Red Kangaroo)	Hunted in NSW, QLD and SA for commercial purpose	0% (0/5)	Blackall, Queensland	No specified time	Mouse bioassay	Smith and Munday, (1965)	https://doi.org/10.1111/j.1751-0813.1965.tb06562.x
		0% (0/6)	Longreach, Queensland	No specified time	Mouse bioassay	Smith and Munday, (1965)	https://doi.org/10.1111/j.1751-0813.1965.tb06562.x
*Macropus rufogriseus* (Bennett's Wallaby)	Hunted in Tasmania for commercial purpose	0% (0/1)	Tasmania	No specified time	Sabine-Feldman dye test	Munday, (1972)	https://doi.org/10.7589/0090-3558-8.2.169
		3.3% (5/151)	Tasmania	No specified time	ELISA	Johnson et al. (1988)	https://doi.org/10.1111/j.1751-0813.1988.tb14456.x
		8% (2/25)	Tasmania	No specified time	Modified agglutination test (not IGM)	Hollings et al. (2013)	https://doi.org/10.1016/j.ijppaw.2013.02.002
*Thylogale billardierii* (Pademelon)	Hunted in Tasmania for commercial purpose	42.9% (3/7)	Tasmania	No specified time	Sabine-Feldman dye test	Munday, (1972)	https://doi.org/10.7589/0090-3558-8.2.169
		17.7% (15/85)	Tasmania	No specified time	ELISA	Johnson et al. (1988)	https://doi.org/10.1111/j.1751-0813.1988.tb14456.x
		12.3% (28/228)	Tasmania	No specified time	Modified agglutination test (not IGM)	Hollings et al. (2013)	https://doi.org/10.1016/j.ijppaw.2013.02.002
*Macropus agilis* (Agile Wallaby)	Hunted for non-commercial purpose – States not specified	2% (1.26/63)	Northern Australia	No specified time	Autopsy; histological examinations; microscopy	Spear et al. (1983)	https://doi.org/10.1071/WR9830089
*Macropus parryi* (Whiptail Wallaby)	Hunted for non-commercial purpose – States not specified	No literature	–	–	–	–	–
*Petrogale lateralis* (Black Footed Rock Wallaby)	Not hunted	0% (0/26)	South Western Australia	1979	Indirect haemagglutination inhibition test	Jakob-Hoff and Dunsmore, (1983)	https://doi.org/10.1111/j.1751-0813.1983.tb09588.x
*Setonix brachyurus* (Quokka)	Not hunted	4.3% (4/92)	Rottnest Island, Western Australia	11.1961	Histopathology (left lateral femoral muscle biopsy)	Gibb et al. (1966)	https://doi.org/10.1038/ivb.1966.63
		70% (14/20)	Rottnest Island, Western Australia	11.1963	Histopathology (left lateral femoral muscle biopsy)	Gibb et al. (1966)	https://doi.org/10.1038/icb.1966.63
		7.1% (2/28)	Rottnest Island, Western Australia	11.1963	Mouse bioassay (suspension of host skeletal muscle; injected IP; histopathological examination of mice)	Gibb et al. (1966)	https://doi.org/10.1038/icb.1966.63
				–			
				02.1964			

On November the 18th 2020, the Australian government published a new and revised version of the commercial code by AgriFutures Australia. In addition to the six species described above, *Macropus eugenii* is added as a 7th species hunted for commercial purpose.

### 4.3 Handling of food-borne pathogens and food security checks in Australia

Grey literature revealed that checks against food-borne pathogens is not a part of the official *Australian New Zealand Food Standard Code*. This was revealed by an official *Written Question on Notice*, answered at the *Supplementary Budget Estimates 2013–14* on November 20th, 2013 by senator Rhiannon (the official question code is E13-196). The unknown questioner pointed out, that raw kangaroo meat is not checked for food-borne pathogens such as *T*. *gondii* or *Salmonella* spp., because *this is not a requirement of the Australian New Zealand Food Standards Code* and asked, if this is still the case. The question was answered with yes. Food-borne pathogens as well as the checks for them are not part of the official *National Codes of Practice for the Humane Shooting of Kangaroos and Wallabies* (Senate Community Affairs Committee, 2013). The official statement is online available (see reference list).

Based on the reviewed data on seroprevalence, it can be assumed that the majority of the macropods that are hunted have a high prevalence of *T*. *gondii*. However, the impact of this on human health is neglected in the scientific literature as well as in official governmental codes and laws. The high seroprevalence of macropods as well as the genetic diversity of *T*. *gondii* has the potential to become a serious public health threat. Outbreaks in other countries, related to the consumption of undercooked food have already been attributed to a possible genetic diversity of *T*. *gondii* (Schumacher et al., 2020). Furthermore, outbreaks of toxoplasmosis, associated with undercooked kangaroo meat have been already reported in the past. In 1995, an outbreak of toxoplasmosis with 12 cases, including one case of congenital transmission, was likely associated with the consumption of rare kangaroo meat (Robson et al., 1995). Nevertheless, official food checks are still not part of the official Food Security Codes in Australia.

Kangaroo meat is not processed and readily available after the hunt. Cats usually love the meat and their owners buy it in large bags in animal supply stores. Those stores assure their costumers that the meat is safe and clean, although this statement is not reliable due to a lack of controls. Sometimes, the owners also feed kangaroo meat bought in supermarkets or at butchers (Sphynxlair, 2013). 3000 tons of kangaroo meat is exported annually to 60 countries. 75% of this meat is used for pet food (Kenyon, 2019). While frozen meat is safe because the cold kills the microbes, it is not uncommon for cat owners to feed their cats raw, fresh meat. It is recommended to freeze the meat for 2 day at −12°. The meat should not be thawed at room temperature (Wilson 2021). This consumption will potentially increase the risk of infection of cats and spread of *T*. *gondii* oocysts in the environment. This inturn will increase the risk for macropods to become infected and has the potential to create a vicious cycle in which the seroprevalence in cats and kangaroos is mutually increased. Due to the lack of wild feline species in Australia, pet and stray cats are the only definitive hosts for *T*. *gondii* in Australia.

It is very likely that Australia will extend its kangaroo hunting industry. This hypothesis is supported by the expansion of the permitted hunted species from six to seven in November 2020. The new species that was added, *M*. *eugenii*, shows also seroprevalence of *T*. *gondii* (Taggart et al., 2019). This has the potential to increase the risk of outbreaks associated with undercooked kangaroo meat. The majority of bunted kangaroo meat is exported fresh to other countries. Today, the meat is exported to more than 60 countries. Because of this, the risk does not only affect Australia and could have implications for trade (Wilson and Edwards, 2019). However, the meat is exported frozen, which significantly reduces the risk. On the other hand, this frozen meat is less well received by customers than non-frozen meat. This could lead to a conflict between health and economic aspects (Lambooij et al., 2019).

### 4.4 Prevention and treatment implications

There are several approaches to minimise the risk of infection of *T*. *gondii*. However more research is needed in this field to gain robust scientific evidence. Some considerations that scientists should take into account in their research have already been described (Hillman et al., 2016). Researchers must think about the validation of the used diagnostic tests. Other tests should be used to confirm the results and to rule out false positives or false negatives. For example, Parameswaran et al. used PCR to verify their ELISA results (Parameswaran et al., 2009a, 2009b). Researchers should plan their research with a sample size which is big enough to make justifiable conclusions. While Pan et al. examined the organs of infected macropod species and detected a genetic diversity with only 16 examined individuals, their sample size was quite small making definitive conclusions difficult (Pan et al., 2012). Other publications that describe seroprevalence often rely on small sample sizes (Moré et al., 2010; Luo et al., 2017). Sampling strategies are another source of biases. Taggart et al. describe inherent limitations and biases which influence their work. One was the collection of road-killed animals for the examination. They describe, that the true seroprevalence of road-killed animals may be underestimated due to the collection on the basis of convenience and not randomly (Taggart et al., 2019). Other considerations described by Hillman et al. are the performing of cohort studies, the controlling of confounding variables and the potential influence of data clustering (Hillman et al., 2016). In addition to these points, researchers should also think to include a one health approach in their research. In summary, more rigorously designed research is required to gain a deeper understanding of seroprevalence of *T*. *gondii* in Australian macropods. This includes using tests with greater sensitivity and including larger sample sizes in the studies that are randomly selected.

If people consume kangaroo meat, they should be sure that the meat is well cooked. Cats should not feed with fresh meat. Safer alternatives are dry or canned food (Robson et al., 1995; Yücesan et al., 2019). Part of this approach is also the introduction of people education and health promotion. In Australia, education about *T*. *gondii* is neither routinely nor systematically undertaken (Robson et al., 1995). The health impacts of consuming kangaroo meat for humans and pets requires further research. By more clearly understanding the risks, appropriate health education messages and broader health promotion initiatives can be developed to protect the health of pets and humans who consume kangaroo meat.

Checks of kangaroo meat should be introduced to check not only for *T*. *gondii* but also for other food-borne pathogens routinely. Currently, routinely food checks are not part of the official food standards in Australia or in New Zealand. An ELISA can be a useful method to detect the seroprevalence. A PCR is useful to verify the results of these screenings and rule out false positive results (Parameswaran et al., 2009a, 2009b). There are several different methods to detect *T*. *gondii* in food. Bioassays are the established reference method for the isolation of *T*. *gondii* from food. However, those assays require the use of living animals and are very time consuming. For these reasons, bioassays are not recommended for food security checks, slaughterhouse testing or monitoring of commercial meat products (Andreoletti et al., 2007). PCR or Real-time PCR are more recommended methods. In studies, PCR were able to detect parasite contamination (for example 5 × 10^3 trophozoites/g in one study). However, the high salt content of some cured meat limits the sensitivity of PCR assays by inhibiting of the polymerase enzyme and reducing the sensitivity of tissue culture due to osmotic pressure (Warnekulasuriya et al., 1998; Andreoletti et al., 2007).

## 5 Conclusion

This literature review has identified positive seroprevalence of *T*. *gondii* in Australian macropods which could have health implications related to human and pet consumption of kangaroo meat. Further research is required to develop a clearer understanding of these risks and findings can be used to inform policies related to food standards and checking of kangaroo meat as well as guidelines regarding screening for toxoplasmosis in high risk groups such as pregnant women.

## Limitations

This review is influenced by multiple limitations. In reviews, the use of the most appropriate databases is crucial to identify as many publications as possible. This review is limited to publications, listed in Medline, Web of Science, SCOPUS and Informit. Although these four databases cover a wide range of publications, it is possible some publications were not identified.

## Funding

This review received no external funding.

## Declaration of competing interest

The author state that there is no conflict of interest.
